# Work engagement and workers’ health, is there any connection in the social work in ukraine?

**DOI:** 10.1192/j.eurpsy.2021.1227

**Published:** 2021-08-13

**Authors:** A. Dorokhina, O. Chaban, O. Karagodina

**Affiliations:** 1 Department Of Medical Psychology, Psychosomatic Medicine And Psychotherapy, O.O.Bogomolets’ National Medical University/National Children’s Specialized Hospital “OKHMATDYT” of Ministry of Health of Ukraine, Kyiv, Ukraine; 2 Department Of Medical Psychology, Psychosomatic Medicine And Psychotherapy, O.O.Bogomolets’ National Medical University, Kyiv, Ukraine; 3 Social Work And Applied Psychology Department, Academy of Labour, Social Relations and Tourism, Kyiv, Ukraine

**Keywords:** work engagement (WE), socio-demographic profile, social workers (SW), health

## Abstract

**Introduction:**

This study is a part of the bigger research project on the burnout syndrome risk and prevention factors [1,2,4].

**Objectives:**

At the current phase we aimed to discover: 1- trends observed in sociodemographic profiles of Ukrainian social workers(SW) who respond to the online survey; 2- if there is any correlation between the SW work engagement(WE) and general health(H).

**Methods:**

The survey is designed out of two questionnaires - Gallup Q12 Employee Engagement survey(Q12) and the 15-item Patient Health Questionnaire(PHQ-15). Questions on the socio-demographic status are included according to the study purpose [3,4]. SW ‘from the field’ in Ukraine included in the study group(SG). Other professionals (doctors, lawyers, etc.) formed the comparison group(CG). Descriptive statistics applied for the data analyses.

**Results:**

Our sample has the next socio-demographic characteristics: age 20-57 (average 33.2) years old, male/female ratio is 0.36; single at the moment of the study are 66.7%. The SG: women-87.5%, married-62.5%, social work experience -from 1 to 15years. In 28.6% of the CG respondents the Q12 revealed low(less than 50%) WE while in the SW no one showed low WE. PHQ-15: in the SG -62.5% mild and 12.5% -severe somatic problems; in the CG -57.1% mild and 28.6% -severe somatic symptoms. There were no statistically significant differences between 2 groups with regard to WE and H (p<0.05). Relations between variables are non-linear; therefore,Spearman’s coefficient (ρ) applied.

**Conclusions:**

The weak association between the work engagement and health condition (ρ=0.3;p<0.05) is found. There are several limitations due to the sample specificity (online users in Ukraine).This study is ongoing.

